# Antimicrobial Use and Epidemiological Resistance Profiles of Commensal *Escherichia coli* from Swine Farms in Córdoba, Argentina

**DOI:** 10.3390/antibiotics15010086

**Published:** 2026-01-15

**Authors:** Nicolás Javier Litterio, María del Pilar Zarazaga, Augusto Matías Lorenzutti, Juan Pablo Vico, Martín Alejandro Himelfarb, Mariano Guillermo Tinti, Ana Paola Zogbi, Sonia Rubio-Langre, Manuel Ignacio San Andrés Larrea

**Affiliations:** 1 Facultad de Ciencias Agropecuarias, IRNASUS CONICET-UCC, Universidad Católica de Córdoba, Av. Armada Argentina 3555, Córdoba X5016DHK, Argentina; pzarazaga@ucc.edu.ar (M.d.P.Z.); matiaslorenzutti@ucc.edu.ar (A.M.L.); juanpablo.vico@ucc.edu.ar (J.P.V.); martinhimelfarb@ucc.edu.ar (M.A.H.); mariano.tinti@ucc.edu.ar (M.G.T.); zogbi.ana@ucc.edu.ar (A.P.Z.); 2 Department of Pharmacology and Toxicology, Faculty of Veterinary Medicine, Universidad Complutense de Madrid, 28040 Madrid, Spain; sonrubio@ucm.es

**Keywords:** antimicrobial use, multidrug resistance, swine production

## Abstract

Background/Objectives: The expansion of intensive swine production in Córdoba, Argentina, underscores the need to assess the risks associated with antimicrobial (AM) use, whose extensive application has driven antimicrobial resistance, a major global threat within the One Health framework. This study aimed to characterize AM use practices and evaluate the epidemiological resistance profiles (non-wild-type phenotypes, NWT) of commensal *Escherichia coli* of fecal origin from swine farms, using epidemiological cut-off values (ECOFFs) as a surveillance criterion. Methods: An observational cross-sectional study was conducted in 19 farrow-to-finish farms in Córdoba during 2023. Information on AM use (prophylaxis, metaphylaxis, treatment) across production categories was collected. A total of 437 *E. coli* isolates were obtained from fecal samples, and minimum inhibitory concentrations (MICs) were determined for 10 AMs of critical importance for human and animal health. NWT phenotypes were classified according to EUCAST ECOFFs, and multidrug resistance (MDR) was assessed. Results: AM use was frequent and predominantly prophylactic (89.5% of farms), mainly through mass medication (66.3%), with macrolides and amoxicillin being the most commonly administered AMs. NWT proportions were extremely high (90–92%) for ampicillin, tetracyclines, and chloramphenicol and substantial for ciprofloxacin (50.6%), sulfamethoxazole (68.2%), and trimethoprim (44.9%). Extended-spectrum β-lactamase (ESBL)-producing phenotypes were detected. Alarmingly, 92% of isolates were classified as MDR *E. coli*, with homogeneous distribution across production categories. Conclusions: Findings reveal intensive and largely empirical AM use that has consolidated a stable intestinal resistome in the swine population. High MDR levels, even in categories with limited direct AM exposure or involving banned compounds, suggest that co-selection and horizontal gene transfer are key drivers of resistance. This scenario highlights the urgent need to strengthen integrated surveillance and promote prudent AM use strategies under the One Health approach to preserve therapeutic efficacy.

## 1. Introduction

The Argentine Republic, traditionally recognized for its beef production, has experienced a remarkable expansion of the swine industry over recent decades, establishing this sector as a key component of both regional and national economic development. In 2024, swine slaughter reached a historic record of 8.3 million heads, with a total production exceeding 785,000 tons of pork, representing a 6% increase in volume compared with the previous year. The province of Córdoba stands out as one of the country’s main swine-producing regions, contributing 17% of the national slaughter, second only to Buenos Aires [1].

Swine production systems in Córdoba are characterized by their intensive management approach, which demands strict environmental and nutritional control to optimize productivity and reduce sanitary risks [2]. However, even under these conditions, significant animal health challenges persist, strongly influenced by the extensive use of antimicrobials (AMs) for therapeutic, metaphylactic, and prophylactic purposes—agents that remain essential tools in veterinary medicine [3,4]. The indiscriminate or empiric use of these drugs has contributed to the emergence and dissemination of AM resistance, widely recognized as one of the greatest global threats to health [5].

The expansion of AM resistance compromises the effectiveness of treatments in animals and poses a significant threat to public health and the environment [5,6]. Because resistant bacteria and resistance determinants can be transferred among animals, humans, and the environment, the One Health framework underscores the need for coordinated, cross-sectoral strategies [7]. According to the European Food Safety Authority (EFSA), harmonized monitoring of indicator bacteria in food-producing animals enables the assessment of temporal trends, regional comparisons, and the development of evidence-based interventions [8]. This approach relies on standards such as those proposed by the Clinical and Laboratory Standards Institute (CLSI) and the European Committee on Antimicrobial Susceptibility Testing (EUCAST), which provide uniform methodologies and increase the reliability of minimum inhibitory concentration (MIC) distribution assessments—a fundamental basis for the epidemiological surveillance of resistance [9,10].

Despite the global relevance of this issue, specific studies addressing the emergence of bacterial resistance in swine production in Córdoba are scarce. This gap limits surveillance efforts and hampers the development of evidence-based policy. In this context, the aim of this study was to characterize AM-use practices in intensive swine-production systems in Córdoba and to evaluate MIC distribution profiles for AMs of critical importance for public and animal health in fecal *Escherichia coli* isolates obtained from these farms. Epidemiological cut-off values (ECOFFs) were used to distinguish wild-type (WT) from non-wild-type (NWT) isolates, a key criterion for monitoring the emergence of AM resistance. The findings seek to provide scientific evidence to strengthen regional epidemiological surveillance and to support the development of comprehensive strategies to mitigate AM resistance.

## 2. Results

### 2.1. Antimicrobial Use Practices

**Figure 1:**
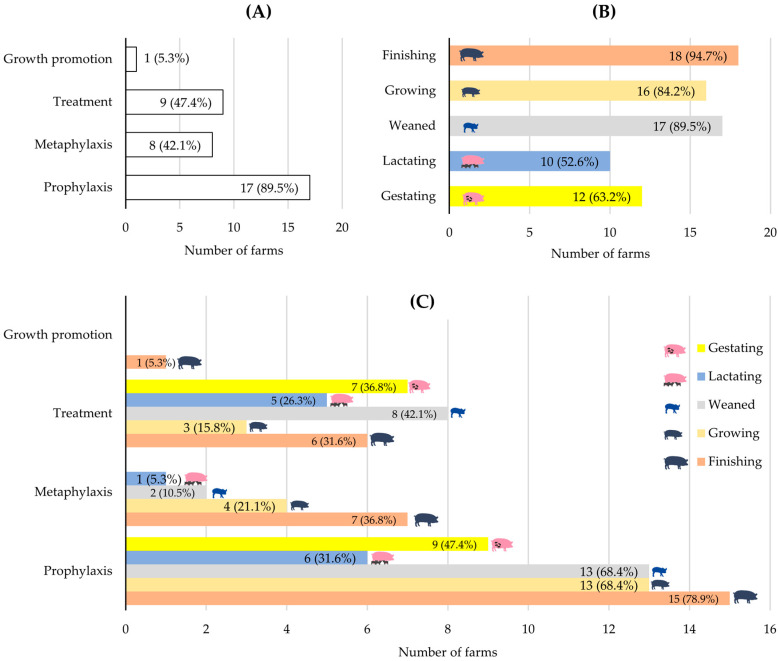
Number of farms (*n*; %) that reported antimicrobial use according to (**A**) purpose of use, (**B**) swine production categories, and (**C**) combination of purpose of use and production categories. Percentages were calculated over the total number of participating farms (*n* = 19). The lactating category included sows and their suckling piglets (≤28 days). Prophylaxis and growth promotion were considered routine practices, whereas metaphylaxis and treatments were recorded for the 30 days prior to the survey.

As shown in Figure 1A, 89.5% of farms reported using AMs for prophylactic purposes. During the 30 days preceding the survey, 47.4% had used AMs for therapeutic treatments, and 42.1% reported metaphylactic use. Additionally, only one farm reported the use of AMs for growth-promotion purposes. According to Figure 1B, regardless of the intended purpose, the finishing phase accounted for the highest AM use (94.7% of the nineteen farms), followed by the weaning and growing phases, reported by 89.5% and 84.2% of farms, respectively. In contrast, the category with the lowest proportion of farms reporting AM use was the lactating phase (52.6%). In this category, AM administration was mainly directed at sows housed with their litters, primarily due to postpartum respiratory, digestive, or reproductive conditions.

Integrating this information, the distribution of farms using AMs across production categories according to the purpose of use (Figure 1C) shows that prophylaxis was most frequent during the finishing phase (78.9%), followed by the growing and weaning phases, each reported by 68.4% of farms. Metaphylaxis also predominated in the finishing phase (36.8%), whereas therapeutic treatments were mainly directed at weaned pigs (42.1%) and gestating sows (36.8%).

Most farms (*n* = 12) housed 500 or more sows, reflecting larger production scale (Table 1). Metaphylaxis was reported by 71.4% of farms with fewer than 500 sows, compared with 25% of those with 500 or more, yielding an OR = 7.5 (*p* < 0.05). In contrast, prophylaxis and therapeutic treatment showed inverse associations (OR = 0.55 and 0.29, respectively), although without statistical significance (*p* > 0.05). Overall, metaphylaxis was more frequent in smaller-scale farms, whereas larger farms tended to favor prophylactic and therapeutic AM use, although without statistically significant differences.

**Figure 2:**
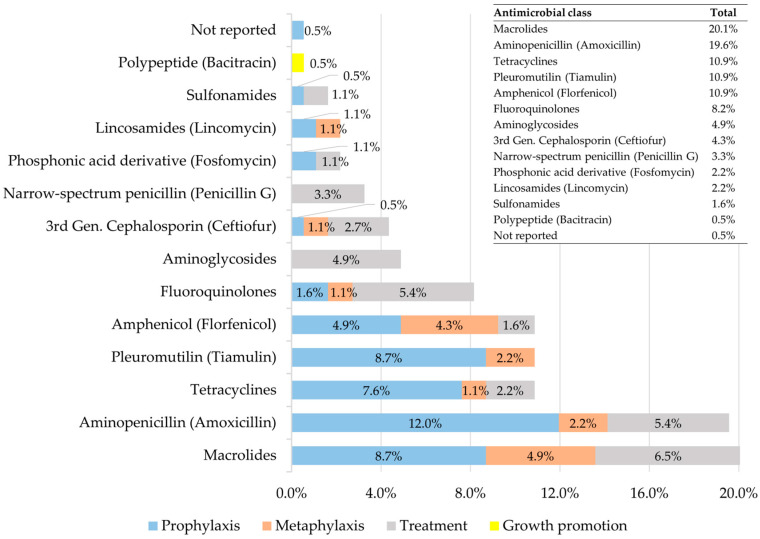
Antimicrobials used and their proportions according to intended purpose (prophylaxis, metaphylaxis, treatment, and growth promotion) in 19 swine production farms. The total number of recorded cases (*n* = 184) includes the different animal categories. Further details are provided in Table S2. Macrolides: tylosin, tilmicosin, tulathromycin, tildipirosin, and erythromycin; Tetracyclines: chlortetracycline, doxycycline, and oxytetracycline; Fluoroquinolones: enrofloxacin and norfloxacin; Aminoglycosides: streptomycin and gentamicin; Sulfonamides: sulfamethazine and sulfamethoxazole combined with trimethoprim.

Figure 2 shows the frequency of use of the different AM classes according to the intended purpose. In total, information was recorded for 22 individual AMs used on 184 occasions across the 19 surveyed farms, although one of them provided only partial data. Complete details are presented in Table S1. The most frequently used were macrolides (20.1%) and amoxicillin (19.6%), followed by tetracyclines, tiamulin, and florfenicol (10.9% each), and fluoroquinolones (8.2%). All remaining AMs/classes were used in less than 5% of recorded cases.

In prophylaxis, the most commonly used AMs were amoxicillin (12.0%), macrolides and tiamulin (8.7% each), and tetracyclines (7.6%). In metaphylaxis, macrolides (4.9%) and florfenicol (4.3%) stood out. Therapeutic treatments showed a more diverse pattern, led by macrolides (6.5%), amoxicillin and fluoroquinolones (5.4% each), and aminoglycosides (4.9%), mainly streptomycin combined with penicillin G. For growth promotion, bacitracin was the only AM reported (0.5%). Other relevant AMs included ceftiofur (4.3%) and fosfomycin (2.2%), primarily used for therapeutic purposes. In one instance, the AM used was not reported (0.5%).

Table S1 provides detailed information on individual AMs used. Among macrolides, tylosin and tilmicosin were the most common, followed by tulathromycin, tylvalosin and erythromycin. Within tetracyclines, chlortetracycline predominated, while enrofloxacin and norfloxacin were the fluoroquinolones employed. Amoxicillin was recorded across all production categories, mainly in weaned and growing pigs. Tiamulin and florfenicol were most frequently used during the finishing phase.

AM selection was based on clinical or epidemiological criteria, and only three surveys mentioned the use of culture and susceptibility testing to guide therapeutic or metaphylactic decisions. The conditions treated through AM therapy and/or metaphylaxis primarily affected the respiratory (34.3%) and digestive (27.1%) systems, followed by the nervous, reproductive, and locomotor systems (<6.5%). Notably, in 28.1% of cases, the affected system treated with AMs was not reported.

Regarding administration, AMs were delivered either individually via injectables or as group administration through feed, water, or even injectables, using various dosing regimens (Table 2). These regimens included daily dosing at constant frequencies (continuous), limited-duration dosing, or cyclical administration with rest periods (intermittent). Limited-duration dosing ranged from 1 to 7 days for injectables (both mass and individual) and from 7 to 15 days for mass administration through feed or water. Group administration predominated (66.3%), distributed mainly between prophylaxis (47.3%) and metaphylaxis (17.9%). Continuous exposure occurred in 16.3% of cases, corresponding to group administration through feed, while the remaining cases varied according to the duration of intermittent or limited regimens. It should be noted that in 25% of cases the daily dosing scheme was not reported, mainly for individual injectable administrations. Complete information for each AM is available in Table S1.

### 2.2. Epidemiological Resistance Profiles

From 450 fecal samples, 437 *E. coli* isolates were obtained (93 from gestating sows, 84 from lactating animals, 79 from the weaning phase, 92 from the growing phase, and 89 from finishing pigs).

Isolates were classified using the epidemiological cutoff values (ECOFFs) established by EUCAST [10], in order to distinguish wild-type (WT) microorganisms from non-wild-type (NWT) ones, indicating absence or presence of acquired resistance, respectively (see detailed methodology in Section 4, Materials and Methods).

**Figure 3:**
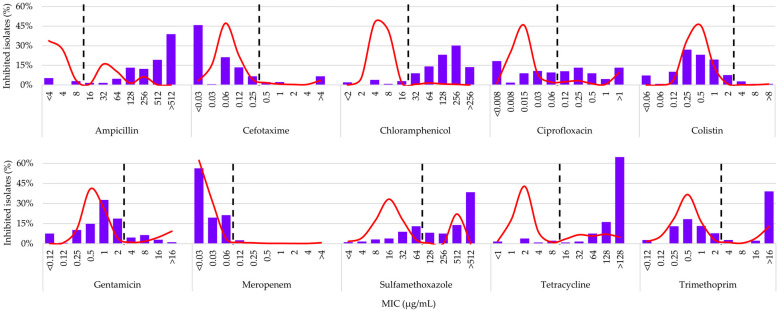
MIC distributions (blue bars) of antimicrobials against commensal *Escherichia coli* (*n* = 437) isolated from feces of swine farms (*n* = 19) in Córdoba, Argentina. The solid red line in each panel represents the reference MIC distribution from the European Committee on Antimicrobial Susceptibility Testing (EUCAST). The vertical dashed black line indicates the epidemiological cutoff value (ECOFF) according to EUCAST; for sulfamethoxazole, the tentative ECOFF was calculated using ECOFFinder v.2.1 [11].

The MIC distribution profiles of the evaluated AMs are shown in Figure 3, illustrating the shape of the local distributions alongside the corresponding epidemiological reference curves reported by EUCAST [10]. Complementarily, Table 3 summarizes the relative inhibition frequencies for each concentration, providing the numerical detail associated with these distributions.

Clear MIC shifts toward higher values were observed for ampicillin (AMP), tetracycline (TET), chloramphenicol (CHL), sulfamethoxazole (SMX), and trimethoprim (TMP), with NWT modes at >512, >128, 256, >512, and >16 µg/mL, respectively, and NWT proportions of 90–92% (AMP, TET, CHL), 68.2% (SMX), and 44.9% (TMP). For ciprofloxacin (CIP), the NWT proportion reached 50.6%, with a broad distribution across multiple concentration levels. Conversely, gentamicin (GEN), cefotaxime (CTX), colistin (COL), and meropenem (MEM) exhibited distributions largely dominated by WT isolates (85–97%). In GEN, MICs increased up to a modal value of 1 µg/mL, close to the ECOFF (2 µg/mL); in COL, a homogeneous distribution between 0.25 and 1 µg/mL predominated; and in CTX and MEM, MIC distributions shifted toward lower values.

Among the isolates exhibiting NWT phenotypes to CTX (*n* = 53; 12.1%), 34 expressed an extended-spectrum β-lactamase (ESBL) phenotype, 9 an AmpC phenotype, 6 showed concurrent ESBL and AmpC phenotypes, and 4 tested negative for both mechanisms. As summarized in Table 4, ESBL-positive isolates were predominantly associated with *blaCTX-M*, detected either alone or in combination with other β-lactamase genes. Overall, *blaCTX-M* was detected in 29 isolates, whereas *blaTEM* was identified in 18 isolates, either as the sole determinant or in combination with *blaCTX-M*. In contrast, *blaSHV* was not detected in any isolate. Notably, one *blaTEM*-positive isolate exhibited a non-ESBL phenotype, a finding consistent with the presence of a non-ESBL TEM variant.

**Figure 4:**
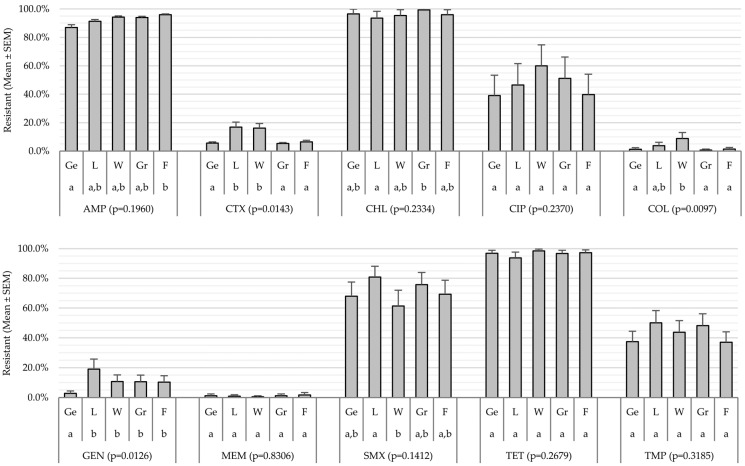
Resistant *Escherichia coli*, categorized by production stage. AMP: Ampicillin; CTX: Cefotaxime; CHL: Chloramphenicol; CIP: Ciprofloxacin; COL: Colistin; GEN: Gentamicin; MEM: Meropenem; SMX: Sulfamethoxazole; TET: Tetracycline; TMP: Trimethoprim. Ge: Gestating; L: Lactating; W: Weaned; Gr: Growing; F: Finishing. *p*-value indicates overall significance (*p* < 0.05). Identical letters (“a” or “b”) indicate that the means ± SEM for the same antimicrobial do not differ significantly among production stages.

Analysis by production category revealed significant global effects (*p* < 0.05) for GEN, CTX, and COL (Figure 4). NWT proportions for GEN were lowest in gestating animals (2.8 ± 1.6%), whereas lactating and weaning categories showed higher values for CTX (16.9 ± 3.4% and 16.2 ± 3.2%, respectively), and the weaning phase also showed the highest proportion for COL (8.8 ± 4.3%). No global effects were detected for the remaining AMs, although some pairwise differences were observed: AMP was higher in finishing than in gestation, CHL was higher in the growing phase than in lactating animals, and SMX was higher in lactating animals than in the weaning category.

Multidrug resistance (MDR) was also evaluated according to the criteria described in Section 4 (Materials and Methods). In total, 402 isolates (92%) were classified as MDR *E. coli*, while 33 isolates (7.5%) showed an NWT phenotype to one or two AMs classes, and only two isolates (0.5%) were fully WT (Table 5). MDR frequency was homogeneously distributed across production categories. Most isolates exhibited NWT phenotypes to four (34.1%) or five (34.8%) AMs classes. MDR *E. coli* were detected in all farms evaluated (19/19), and the most frequent combination of affected classes was AMP + TET + CHL + CIP + SMX, identified in 59 isolates from 11 farms (Table S2). Four isolates were NWT to all evaluated classes, although none were NWT to all 10 Ams tested, as all isolates remained WT to MEM, and only three remained WT to CTX.

## 3. Discussion

### 3.1. Antimicrobial Use Practices: Interpretation and Implications

The use of AMs in the swine farms included in this study was largely dominated by prophylaxis (89.5%), particularly during the finishing phase (78.9%), a critical stage due to the high risk of respiratory and digestive diseases in intensive systems. High preventive use was also observed during the weaning and growing phases (68.4%), consistent with studies describing these stages as especially vulnerable to stress, dietary transitions, and rapid growth [12].

Metaphylaxis was reported in 42.1% of farms, occurring most frequently during finishing (36.8%). This pattern aligns with reports highlighting its role in controlling respiratory and digestive outbreaks under conditions of high animal density [13]. In our study, metaphylaxis was more common in smaller-scale farms (OR = 7.5; *p* < 0.05), in agreement with observations linking this practice to structural and economic limitations that increase reliance on AMs [14].

Therapeutic use was reported in 47.4% of farms, primarily in weaned pigs (42.1%) and gestating sows (36.8%). This pattern reflects the increased susceptibility of the weaning phase to gastrointestinal and respiratory infections due to immunological immaturity, dietary change, and stress. In gestating sows, the use of AMs reflects susceptibility to reproductive disorders, mastitis, and locomotor problems associated with intensive management [15]. The main indications for both treatment and metaphylaxis were respiratory (34.3%) and digestive (27.1%) diseases, although in 28.1% of cases the affected system was not reported; the prominence of respiratory conditions as a driver of AM use is consistent with post-weaning disease patterns [16].

Regardless of the purpose of use, the lactating category showed the lowest use of AMs (52.6%), directed mainly toward sows for postpartum respiratory, digestive, or reproductive conditions. The low use in suckling piglets is explained by the protection conferred by colostrum and milk [3], whereas postpartum disorders in sows remain a recurrent health challenge [15].

The use of AMs as growth promoters was exceptional, detected in only one farm (bacitracin), in line with the transition toward their elimination and the recent prohibition of this practice in Argentina [17], consistent with the global trend reported by the World Organisation for Animal Health (WOAH) [18].

Macrolides were the most frequently used AM class (20.1%), with participation in prophylaxis, metaphylaxis, and treatments. This predominance reflects their central role in the control of respiratory and digestive infections in intensive systems, consistent with international reports [3]. Within this class, tylosin and tilmicosin were the most commonly used, particularly during the finishing phase, where major respiratory health challenges are concentrated [19,20,21]. Other macrolides, such as tulathromycin and tylvalosin, were employed less frequently, consistent with their use in specific prophylactic or therapeutic contexts [22,23].

As an individual AM, amoxicillin was the most widely used (19.6%), mainly for prophylactic purposes during weaning and growing. This pattern aligns with its extensive use in preventing post-weaning diarrhea and bacterial pneumonias [12]. Treatments with amoxicillin were consistent with its indications for pneumonias and colibacillosis in pigs [15]. Group administration via feed was the predominant route, with highly variable dosing regimens across farms (Table S1). Although this practice is common due to its efficiency in large groups, it is discouraged for prolonged use without microbiological diagnosis because of the risk of selecting NWT phenotypes [18].

Tetracyclines, tiamulin, and florfenicol each accounted for 10.9% of total AM use, with a predominance during the finishing phase. Tetracyclines, particularly chlortetracycline, were used mainly for group prophylaxis (Table S1), consistent with their documented use to prevent respiratory outbreaks [24], although other studies report greater use during the growing and weaning phases [3,23]. Similarly, tiamulin was primarily used for prophylaxis during finishing, in line with its efficacy against digestive and respiratory infections [25]. In the case of florfenicol, its application was concentrated on respiratory diseases, consistent with previous reports [26], and it also showed complementary use in digestive conditions—a pattern less frequently described in the literature and possibly associated with local sanitary characteristics.

Fluoroquinolones, ceftiofur, and fosfomycin are considered AMs of critical and highest priority for public health [27]. Their use was variable, with approximate proportions of 8%, 4%, and 2%, respectively. Survey responses reflected prudent use when administered individually for specific treatments, highlighting the more controlled use of ceftiofur and the low proportion of fosfomycin. However, cases of group and preventive use were also recorded, particularly involving norfloxacin—an especially concerning pattern given the importance of fluoroquinolones in human medicine.

The selection of AMs was predominantly empirical, based on clinical or epidemiological criteria, as only three farms reported using culture and susceptibility testing. This practice reflects a common logistical constraint in swine production, where costs and operational limitations restrict evidence-based decision-making [28]. Regarding administration, group use predominated (66.3%), mostly for prophylaxis (47.3%) and metaphylaxis (17.9%), with intermittent or continuous oral dosing regimens. Although intermittent regimens represent a step toward more prudent use, the continued reliance on group and continuous administration constitutes a concerning risk factor for resistance selection.

Taken together, the results demonstrate an intensive and largely preventive pattern of AM use, with limited microbiological justification and a predominance of group administration. These practices, centered on prevention rather than targeted therapy, generate sustained selective pressure on the porcine microbiota, a phenomenon directly reflected in the distribution profiles of NWT phenotypes observed in *E. coli*.

### 3.2. Interpretation of Epidemiological Resistance Profiles

The evaluation of NWT phenotypes in indicator bacteria such as commensal *E. coli* is essential for assessing the selective pressure exerted by AMs in animal production and its potential impact on public health [29]. This sentinel microorganism is particularly useful for monitoring the evolution of epidemiological patterns. In this study, the analysis was based on MIC distributions together with the ECOFFs proposed by EUCAST, an approach that allows the differentiation of WT and NWT populations and serves as a sensitive tool for the early detection of emerging resistance mechanisms and for understanding their population dynamics [30,31].

The levels of NWT phenotypes identified were extremely high (90–92%) for AMP, TET, and CHL, consistent with intensive swine systems worldwide [30,31]. This phenomenon is reinforced by comparing the local MIC distributions with the EUCAST epidemiological reference curves (Figure 3), which clearly show pronounced shifts toward higher MIC values for AMP, TET, and CHL, loss of the WT mode, and dominance of NWT subpopulations—evidence of strong selective pressure. SMX and TMP exhibited transitional patterns, with partial loss or bimodality of the distributions depending on the AM. In contrast, CIP displayed a broad and heterogeneous spread without a clear shift or defined modes, suggesting the coexistence of multiple subpopulations with differing susceptibility levels. Meanwhile, the profiles of GEN, CTX, COL, and MEM retained unimodal shapes centered within WT ranges, with no appreciable shifts relative to the reference, indicating limited selective pressure.

In the case of AMP, the expansion of NWT subpopulations toward higher MIC values corresponds to the extensive use of amoxicillin recorded in the farms, where it was the most frequently used AM. Although no global differences were observed among production categories, the finishing phase showed significantly higher values than gestation, possibly due to the cumulative effect of AM use throughout the production cycle. Co-selection driven by other AMs and the environmental persistence of resistance genes may explain the stability of high NWT levels even in categories with lower direct exposure [32].

The high proportion of NWT isolates to CHL is a relevant finding, given its prohibition in food-producing animals [33]. This NWT proportion may be explained by the presence of *floR*, *cfr*, *fexA*, and *cmlA* genes on plasmids and other mobile elements [34,35], which confer cross-resistance with florfenicol—a widely used AM in the farms included in this study. However, florfenicol use alone does not account for the high proportion of NWT phenotypes, since these were observed across all categories without significant global differences, despite its application being concentrated mainly in the growing and finishing phases (Table S1). This suggests that, in addition to direct pressure from florfenicol, co-selection associated with the use of other frequently applied AMs and the joint mobilization of genes such as *cmlA*, along with complementary mechanisms (efflux pumps or ribosomal modifications), may contribute to the persistence of these NWT phenotypes [8,36]. Furthermore, the widespread use of tiamulin for prophylaxis and metaphylaxis may represent an additional co-selective factor for strains carrying the *cfr* gene. Although the prevalence of *cfr* in *E. coli* is limited [37], this gene confers cross-resistance to phenicols and other AMs [38], meaning that tiamulin use could promote the persistence of NWT phenotypes to CHL.

The high proportion of NWT isolates to TET reflects the selective pressure derived from sustained tetracycline use in the farms. Additionally, the frequent use of macrolides may favor co-selection, as resistance genes for tetracyclines and for AMs of the macrolide–lincosamide–streptogramin group commonly coexist within the conjugative transposons Tn916/Tn1545, which facilitate multidrug resistance and the persistence of these determinants even under reduced selective pressure [39].

For SMX and TMP, the moderately elevated NWT profiles and the poor correspondence with recorded levels of use indicate that resistance is maintained through mechanisms of genetic persistence. The co-localization of *sul1*, *sul2*, and *dfrA* within class 1 integrons, together with other resistance genes, promotes their stable transmission among bacteria, even in the absence of direct selection [40]. The higher proportion of NWT isolates in lactating animals may be related to vertical transmission from sows and to the highly contaminated farrowing environment, conditions that facilitate early colonization and persistence of these determinants.

The MIC profile of *E. coli* for CIP was heterogeneous, with coexistence of WT and NWT isolates. The observed NWT proportion (50.6%) is noteworthy given the critically important status of fluoroquinolones in human and veterinary medicine [27,41]. This proportion, despite the restricted use of the class in the farms, suggests the combination of multiple mechanisms, including chromosomal mutations in *gyrA*, *gyrB*, *parA*, and *parC*, together with plasmid-mediated determinants such as *qnr*, *aac(6′)-Ib-cr*, and efflux pumps. These mechanisms allow resistance to persist even without direct exposure, indicating a process of stable fixation within the porcine microbiota [42,43,44].

In aminoglycosides, the low proportion of NWT isolates to GEN is consistent with their limited use. The differences observed among production categories, with lower NWT proportions in gestating animals, reflect the impact of active selective pressure and the intestinal ecological context [45]. During gestation, the microbiota tends to be more stable and less pro-inflammatory, reducing the likelihood of amplification of resistant strains [46].

The profiles for CTX showed a predominance of WT populations, which is encouraging given that this third-generation cephalosporin is considered critically important. The detection of ESBL and AmpC phenotypes associated with *blaCTX-M* and *blaTEM* aligns with reports at both the regional [47] and international [30] levels. The use of ceftiofur on farms, together with the selective pressure exerted by other β-lactams, especially amoxicillin, may have favored the emergence of NWT *E. coli* and the activation of endogenous AmpC mechanisms [48]. The higher proportions of NWT isolates in lactating animals and weaners could be related to the physiological stress and microbiome instability characteristic of these stages [2].

For MEM, the presence of only a few NWT isolates, consistent with the prohibition of its use in animals [17,27], suggests possible environmental dissemination of carbapenemase genes, favored by the pressure of other β-lactams and by horizontal transfer [48,49].

Despite the prohibition of COL use in Argentina [50], some NWT isolates were detected. Similar findings have been reported in pigs from northeastern Argentina, including the presence of *mcr-1* [51]. This supports the hypothesis of co-selection of colistin-associated determinants in MDR plasmids, even in the absence of direct use of this AM [52]. Moreover, the higher frequency of NWT in weaners could be related to intestinal instability and stress at this stage [2,23].

Taken together, the high proportion of MDR isolates observed in this study reveals the consolidation of a stable intestinal resistome in the swine population. The simultaneous presence of resistance genes to multiple AM classes—including β-lactams, tetracyclines, phenicols, quinolones, and sulfonamides—suggests the circulation of complex plasmids and integrons that facilitate horizontal transmission and environmental persistence [53]. The homogeneous distribution of MDR across production categories indicates that the dissemination of resistance genes exerts a greater influence than differences in direct exposure. Although susceptibility to CTX and MEM is still largely preserved, the high prevalence of MDR phenotypes underscores the need to strengthen integrated surveillance systems and promote prudent AM use strategies within a One Health framework.

## 4. Materials and Methods

### 4.1. Surveys and Sampling

The study was conducted in swine farms located in the province of Córdoba, Argentina, with prior consent from farm owners. It followed an observational, cross-sectional design, using stratified random sampling according to production category. No animals were handled or subjected to intervention at any stage. Fieldwork was conducted throughout 2023, covering different periods of the year, and included nineteen farrow-to-finish farms operating under intensive production systems, encompassing gestating sows, lactating animals (≤28 days) housed with their mothers, weaned pigs (28–40 days), growing pigs (41–90 days), and finishing pigs (91–180 days).

Information on AM use practices was collected from each farm, disaggregated by production category. A structured survey was administered, gathering data on the purpose of use (prophylaxis, metaphylaxis, treatment, or growth promotion), the AM selected, and the dosing regimen. In addition, the number of breeding sows present on each farm was recorded and later used as a variable to classify herd size. For analytical purposes, prophylaxis and growth promotion were considered routine practices, whereas metaphylaxis and treatments were recorded in relation to the 30 days preceding the survey. This methodological approach followed the recommendations outlined by the WOAH [4].

**Figure 5:**
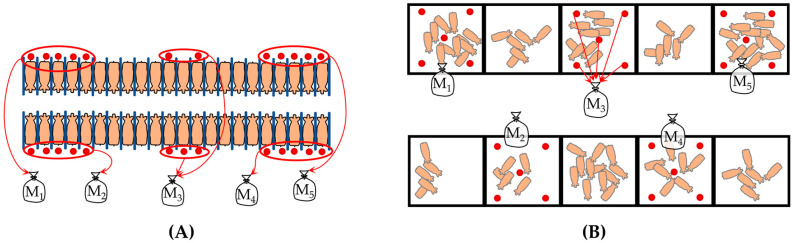
Fecal sampling design for each production unit according to the structure and spatial distribution of the facilities: (**A**) gestating sows and (**B**) other production categories. Red dots indicate the subsamples that make up the composite sample (M).

Complementarily, for each farm and production category, five composite fresh fecal samples were collected from pen floors. Each composite sample consisted of subsamples taken from five different points within the pen using systematic random sampling that accounted for the spatial layout of the facilities (Figure 5). Only freshly deposited feces were sampled in order to minimize environmental exposure and preserve bacterial viability. Samples were stored at 4 °C for no longer than 12 h before being transported to the Veterinary Pharmacology Laboratory at the Faculty of Agricultural Sciences (Universidad Católica de Córdoba), where they were processed. The sampling methodology adhered to EFSA recommendations for resistance studies in indicator bacteria [8].

### 4.2. Bacterial Isolation and Determination of Minimum Inhibitory Concentration

Commensal *E. coli* were isolated and phenotypically identified from fecal samples, following their use as indicator microorganisms in resistance surveillance programs according to WOAH recommendations [29]. Isolation procedures followed ISO 7251 [54]. A 10% *w*/*v* suspension was prepared in sterile peptone water and homogenized in a stomacher (1 min). Tenfold serial dilutions were prepared, and 100 µL were plated onto eosin methylene blue agar. After incubation (37 °C, 24 h), a single metallic green colony exhibiting typical *E. coli* morphology was selected from each sample to minimize the inclusion of duplicate isolates from the same clone and subcultured onto Mueller–Hinton agar. No molecular typing was performed to assess clonal relatedness among isolates. Biochemical identification was performed using indole production, triple sugar iron (TSI), and Simmons citrate tests. Confirmed isolates were stored at −70 °C in brain–heart infusion broth supplemented with glycerol for further analysis.

Subsequently, each *E. coli* isolate was tested against 10 AMs to determine its MIC. The broth microdilution method was used [9], with *E. coli* ATCC 25922 as the control strain. The AMs tested were AMP, CTX, MEM, CIP, TET, GEN, SMX, TMP, CHL, and COL. Serial dilutions were prepared according to CLSI and EUCAST ranges [9,10]. AMs with purity ≥95% *w*/*w* were supplied by Laboratorios OVER SRL (Argentina), except for COL, CIP, and CTX, which were obtained from Sigma-Aldrich (St. Louis, MO, USA).

The selection of AMs followed primarily EFSA recommendations [8], together with those of WHO and WOAH [55,56], prioritizing agents critically important for human health.

Based on the MIC values, microorganisms were classified as WT or NWT to indicate the absence or potential presence of acquired resistance mechanisms, respectively, according to the epidemiological cutoff values (ECOFFs) defined by EUCAST [10]. For SMX, a tentative ECOFF estimated using ECOFFinder v2.1 was employed [11].

Additionally, *E. coli* isolates exhibiting NWT phenotypes to CTX were analyzed using the ESBL + AmpC^®^ kit (Rosco, Albertslund, Denmark) to detect ESBL and AmpC β-lactamases and were subsequently characterized by conventional PCR to identify *blaCTX-M*, *blaTEM*, and *blaSHV* genes. PCR assays were performed using primer sets previously described [57], with minor modifications to the reaction conditions. Amplifications were carried out in a final volume of 25 µL containing 1× reaction buffer, 2 mM MgCl_2_, 200 µM of each dNTP, 0.4 µM of each primer, 1 U of Taq DNA polymerase, and genomic DNA as template. Thermocycling conditions consisted of an initial denaturation at 95 °C for 2 min, followed by 30 cycles of denaturation at 95 °C for 30 s, annealing at 62.5 °C for 30 s, and extension at 72 °C for 1 min, with a final extension at 72 °C for 10 min. A no-template control was included in each PCR run.

### 4.3. Data Analysis

Data on AM use practices were analyzed using descriptive statistics (absolute values and percentages). The association between the number of breeding sows and AM use was evaluated using odds ratios (ORs) with 95% confidence intervals, applying Pearson’s Chi-square test with a significance level of *p* < 0.05.

Epidemiological resistance profiles were characterized by calculating the relative frequency of isolates at each MIC value. These local MIC distributions were visually and descriptively compared with the epidemiological reference distributions published by EUCAST [10].

Differences in the proportion of NWT isolates among production categories were analyzed using a generalized linear mixed model for each AM, with production category as a fixed effect and farm as a random effect. A binomial family with logit link was used (selected based on lower AIC and BIC). Global differences were evaluated using Wald tests, and pairwise comparisons were performed using Fisher’s LSD test (*p* < 0.05).

MDR isolates were quantified and defined as those classified as NWT to at least one agent in three or more AM classes, following adapted criteria from Magiorakos et al. [58]. Seven classes were considered, recognizing that AMP, CTX, and MEM belong to the β-lactam class, and SMX and TMP correspond to folate pathway inhibitors.

All analyses were performed using Infostat v.2020 [59].

## 5. Conclusions

This study provides an integrated overview of AM use and epidemiological resistance profiles in intensive swine production systems in Córdoba, Argentina. The survey revealed frequent and mostly empirical AM use, dominated by prophylactic and metaphylactic schemes. The most commonly employed AMs (macrolides, amoxicillin, tetracyclines, tiamulin, and florfenicol) reflect a sustained pattern of exposure capable of promoting selective pressure and the dissemination of NWT *E. coli* populations.

The phenotypic analysis of commensal *E. coli* showed high proportions of NWT isolates to AMP, TET, and CHL, along with intermediate levels for CIP, SMX, and TMP. These findings indicate the acquisition of NWT phenotypes associated not only with direct AM use but also with co-selection mechanisms and environmental persistence. The detection of NWT isolates to compounds with restricted or prohibited use suggests that resistome dynamics are strongly influenced by horizontal gene transfer rather than by recent antimicrobial exposure.

A high prevalence of MDR *E. coli* was observed across all production categories, confirming the presence of a consolidated intestinal resistome in the swine population. This scenario demands the urgent implementation of integrated strategies under a One Health approach and in alignment with global initiatives aimed at containing AM resistance. Priority actions include the prudent use of AMs, strengthened biosecurity measures, and the sustained development of surveillance and research systems. It is also essential to reinforce professional training and communication strategies directed at producers and the general public to promote the effective adoption of prevention and control practices. These actions are critical to preserving available therapeutic efficacy and ensuring the long-term sustainability of swine production systems.

## Figures and Tables

**Table 1:** Relationship between the number of sows per farm and antimicrobial use.

Purpose of Antimicrobial Use	Farms *n* (%) by Number of Sows		<500/≥500 OR (95% CI)	*p* Value
	<500 Sows *n* = 7 (100)	≥500 Sows *n* = 12 (100)		
Prophylaxis	6 (85.7)	11 (91.7)	0.55 (0.05–6.38)	0.6834
Metaphylaxis	5 (71.4)	3 (25.0)	7.50 (1.09–51.83)	0.048
Treatment	2 (28.6)	7 (58.3)	0.29 (0.04–1.83)	0.2101
Growth promotion	0 (0.0)	1 (8.3)	–	0.4326

OR (95% CI): odds ratio and 95% confidence interval. A *p* value < 0.05 was considered statistically significant.

**Table 2:** Antimicrobial use occasions, expressed as *n* (%), by route and administration type, route, and dosing regimen, according to purpose of use.

Administration (Type/Route/Regimen) *	Prophylaxis	Metaphylaxis	Treatment	Growth Promotion	Total
**Group**	**87 (47.3)**	**33 (17.9)**	**1 (0.5)**	**1 (0.5)**	**122 (66.3)**
*Feed*	*73 (39.7)*	*27 (14.7)*	*1 (0.5)*	*1 (0.5)*	*102 (55.4)*
Continuous	26 (14.1)	3 (1.6)	1 (0.5)	-	30 (16.3)
Intermittent	21 (11.4)	4 (2.2)	-	-	25 (13.6)
Limited	18 (9.8)	20 (10.9)	-	-	38 (20.7)
n.i.	8 (4.3)	-	-	1 (0.5)	9 (4.9)
*Water*	*12 (6.5)*	*3 (1.6)*	*-*	*-*	*15 (8.2)*
Intermittent	2 (1.1)	-	-	-	2 (1.1)
Limited	10 (5.4)	3 (1.6)	-	-	13 (7.1)
*Injectable*	*2 (1.1)*	*3 (1.6)*	*-*	*-*	*5 (2.7)*
Limited	2 (1.1)	3 (1.6)	-	-	5 (2.7)
**Individual**	**-**	**-**	**62 (33.7)**	**-**	**62 (33.7)**
*Injectable*	*-*	*-*	*62 (33.7)*	*-*	*62 (33.7)*
Limited	-	-	25 (13.6)	-	25 (13.6)
n.i.	-	-	37 (20.1)	-	37 (20.1)
**Total**	**87 (47.3)**	**33 (17.9)**	**63 (34.2)**	**1 (0.5)**	**184 (100.0)**

* Type of administration was classified as group or individual. Routes included feed, water, or injection. Regimen (dosing regimen) was defined as follows: Continuous = daily constant use; Intermittent = cyclic administration with withdrawal periods; Limited = short-duration administration (1–7 days for individual or group injectables; 7–15 days for group administration via feed or water); n.i.: not informed.

**Table 3:** MIC distributions of antimicrobials against commensal *Escherichia coli* (*n* = 437). Results are expressed as the proportion (%) of inhibited strains.

Antimicrobial	MIC (µg/mL)																				Cum. % R ^a^	ECOFF (µg/mL)
	<0.008	0.008	0.015	0.03	0.06	0.12	0.25	0.5	1	2	4	8	16	32	64	128	256	512	1024	>1024		
Ampicillin										5.30	0.00	3.00	**1.60**	**1.60**	**4.80**	**13.30**	**12.40**	**19.20**	**38.90**		**91.80**	8
Cefotaxime			45.80	0.70	21.30	13.50	6.60	**2.30**	**2.30**	**0.70**	**0.20**	**6.60**									**12.10**	0.25
Chloramphenicol									2.10	0.00	3.90	0.90	3.00	**8.90**	**14.20**	**23.10**	**30.20**	**13.70**			**90.20**	16
Ciprofloxacin	18.30	1.80	8.90	10.80	9.60	**10.50**	**13.30**	**8.90**	**4.60**	**13.30**											**50.60**	0.06
Colistin				7.30	0.90	10.10	27.00	23.10	19.50	7.60	**2.70**	**0.90**	**0.90**								**4.60**	2
Gentamicin					7.6	0.70	10.30	14.90	32.70	18.80	**4.60**	**6.40**	**3.00**	**1.10**							**15.10**	2
Meropenem			56.30	19.50	21.30	**2.50**	**0.20**	**0.20**	**0.00**	**0.00**	**0.00**	**0.00**									**3.00**	0.06
Sulfamethoxazole										1.10	1.60	3.20	3.90	8.90	13.00	**8.20**	**7.60**	**14.00**	**38.40**		**68.20**	64 **^b^**
Tetracycline								1.60	0.20	3.90	0.90	2.30	**0.90**	**1.60**	**7.60**	**16.20**	**64.80**				**91.10**	8
Trimethoprim					2.70	0.00	13.00	18.30	13.30	7.80	**2.70**	**0.70**	**2.30**	**39.10**							**44.90**	2

(**^a^**) Cumulative % of resistant isolates; (**^b^**) Tentative ECOFF estimated with ECOFFinder v. 2.1 [11]; Non-highlighted percentages correspond to WT isolates, while bold values indicate NWT isolates. Shaded areas represent regions outside the MIC analysis range.

**Table 4:** Distribution of ESBL/AmpC phenotypes and β-lactamase genes among *Escherichia coli* isolates exhibiting NWT phenotypes to cefotaxime (CTX) (*n* = 53).

ESBL/AmpC Phenotype	β-lactamase Genes	*n* (%)
ESBL (+)/AmpC (−)	*blaCTX-M*	13 (24.5)
ESBL (+)/AmpC (−)	*blaCTX-M* + *blaTEM*	11 (20.8)
ESBL (+)/AmpC (−)	*blaTEM*	4 (7.5)
ESBL (+)/AmpC (−)	Negative for *blaCTX-M*, *blaTEM* and *blaSHV*	6 (11.3)
**Subtotal ESBL (+)/AmpC (−)**		**34 (64.2)**
ESBL (+)/AmpC (+)	*blaCTX-M*	3 (5.7)
ESBL (+)/AmpC (+)	*blaCTX-M* + *blaTEM*	2 (3.8)
ESBL (+)/AmpC (+)	Negative for *blaCTX-M*, *blaTEM* and *blaSHV*	1 (1.9)
**Subtotal ESBL (+)/AmpC (+)**		**6 (11.3)**
ESBL (−)/AmpC (+)	Negative for *blaCTX-M*, *blaTEM* and *blaSHV*	9 (17)
ESBL (−)/AmpC (−)	*blaTEM*	1 (1.9)
ESBL (−)/AmpC (−)	Negative for *blaCTX-M*, *blaTEM* and *blaSHV*	3 (5.7)
**Subtotal ESBL (−)**		**13 (24.5)**
**Total**		**53 (100)**

**Table 5:** Number of antimicrobial classes affected (NWT) in *Escherichia coli* isolates and their distribution across production stages (MDR profiles).

Antimicrobial Classes Affected	Isolates *n* (%)	Isolates by Production Stages (*n*)					Farms (*n*)
		Gestating	Lactating	Weaned	Growing	Finishing	
None	2 (0.5%)	1	0	1	0	0	1
One class	9 (2.1%)	1	2	1	1	4	5
Two classes	24 (5.5%)	7	5	3	5	4	10
Three classes	58 (13.3%)	18	14	12	7	7	17
Four classes	149 (34.1%)	33	26	19	35	36	19
Five classes	152 (34.8%)	27	26	30	36	33	19
Six classes	39 (8.9%)	6	9	12	8	4	13
Seven classes ^a^	4 (0.9%)	0	2	1	0	1	3
Total	437 (100.0%)	93	84	79	92	89	19

^a^ Among betalactams, the seven isolates were NWT to ampicillin and one to cefotaxime; all were WT to meropenem. Details of this table can be found in Table S2.

## Data Availability

The data presented in this study are available from the corresponding author upon reasonable request.

## Supplementary Materials

The following supporting information can be downloaded at: https://www.mdpi.com/article/10.3390/antibiotics15010086/s1; Table S1: Antimicrobials used by purpose, animal category, administration route, and dosage in pig farms (*n* = 19) from Córdoba, Argentina.; Table S2: Detailed MDR (NWT) profiles in *Escherichia coli*: combinations of affected antimicrobial classes and agents.
