# Supplementary material for: Antimicrobial Use and Epidemiological Resistance Profiles of Commensal Escherichia coli from Swine Farms in Córdoba, Argentina
Source: Antibiotics (Basel). 2026 Jan 15;15(1):86. doi: 10.3390/antibiotics15010086 (PMC12837807; doi:10.3390/antibiotics15010086)
Supplement: Supplementary file 1 [file antibiotics-15-00086-s001.zip › Table S1.pdf]

Table S1: Antimicrobials used by purpose, animal category, administration route, and dosage in pig farms (n = 19) from Córdoba, Argentina.

	Category	Administration	Dosage	Count: n (%)
**Aminoglycosides**				**9 (4.9%)**
**Streptomycin**				**6 (3.3%)**
Treatment	Gestating	Individual – Injectable	n.i.	2
	Lactating	Individual – Injectable	Dosage n.i.; 2 and 3 days	2
	Weaned	Individual – Injectable	n.i.	1
	Finishing	Individual – Injectable	n.i.	1
**Gentamicin**				**3 (1.6%)**
Treatment	Lactating	Individual – Injectable	n.i.	1
	Weaned	Individual – Injectable	n.i.	1
	Finishing	Individual – Injectable	n.i.	1
**Aminopenicillin (Amoxicillin)**				**36 (19.6%)**
Prophylaxis	Lactating	Group – Injectable	Dosage n.i.; 1 day	1
	Weaned	Group – feed	250 to 500 g/ton feed; continuous	6
		Group – feed	Dosage n.i.; continuous	1
		Group – feed	350 g/ton feed; 7 days	1
		Group – water	20 mg/kg; 7 days	1
		Group – water	Dosage n.i.; 7 days	1
	Growing	Group – feed	200 a 500 g/ton feed; continuous	3
		Group – feed	1000 g/ton feed; continuous	1
		Group – feed	400 g/ton feed; 15/45 days	1
		Group – feed	450 g/ton feed; 10/10 days	1
		Group – feed	350 g/ton feed; 15 days	1
		Group – water	20 mg/kg; 7 days	2
	Finishing	Group – feed	250 g/ton feed; 21/30 days	1
		Group – feed	400 g/ton feed; 15/45 days	1
Metaphylaxis	Weaned	Group – feed	400 and 750 g/ton feed; continuous	2
	Growing	Group – feed	450 g/ton feed; 15 days	1
		Group – feed	20 g/ton feed; 7 days	1
Treatment	Gestating	Individual – Injectable	Dosage n.i.; 7 days	1
	Weaned	Individual – Injectable	5 mg/kg; 3 days	1
		Individual – Injectable	Dosage n.i.; 7 days	1
		Individual – Injectable	n.i.	2
	Growing	Individual – Injectable	Dosage n.i.; 7 days	2
	Finishing	Individual – Injectable	Dosage n.i.; 7 days	1
		Individual – Injectable	n.i.	2
**Amphenicol (Florfenicol)**				**20 (10.9%)**
Prophylaxis	Weaned	Group – water	10 mg/kg; 7 days	1
	Growing	Group – feed	40 g/ton feed; 15 days	1
		Group – feed	40 g/ton feed; 15/15 days	1
		Group – feed	n.i.	1
		Group – water	15 mg/kg; 7 days	1
	Finishing	Group – feed	100 g/ton feed; 10 days	1

		Group – feed	40 g/ton feed; 15/15 days	1
		Group – feed	40 g/ton feed; 15 days	1
		Group – feed	Dosage n.i.; 15 days	1
Metaphylaxis	Lactating	Group – Injectable	Dosage n.i.; 2 days	1
	Growing	Group – feed	20 g/ton feed; 15 days	2
	Finishing	Group – feed	20 g/ton feed; 15 days	2
		Group – feed	40 g/ton feed; 7 days	2
		Group – water	15 mg/kg; 7 days	1
Treatment	Gestating	Individual – Injectable	15 mg/kg; Limited; days n.i.	1
	Finishing	Individual – Injectable	20 mg/kg; 1 day	1
		Individual – Injectable	n.i.	1
**3rd Generation Cephalosporin (Ceftiofur)**				**8 (4.3%)**
Prophylaxis	Weaned	Group – Injectable	Dosage n.i.; 3 days	1
Metaphylaxis	Finishing	Group – Injectable	Dosage n.i.; 3 days	2
Treatment	Gestating	Individual – Injectable	n.i.	1
	Weaned	Individual – Injectable	n.i.	2
	Finishing	Individual – Injectable	Dosage n.i.; 7 days	1
		Individual – Injectable	n.i.	1
**Phosphonic Acid Derivative (Fosfomycin)**				**4 (2.2%)**
Prophylaxis	Gestating	Group – feed	250 g/ton feed; 10/45 days	1
	Weaned	Group – feed	200 g/ton feed; continuous	1
Treatment	Weaned	Individual – Injectable	n.i.	1
	Finishing	Individual – Injectable	n.i.	1
**Fluoroquinolones**				**15 (8.2%)**
**Enrofloxacin**				**10 (5.4%)**
Treatment	Gestating	Individual – Injectable	5 mg/kg; 3 days	1
	Lactating	Individual – Injectable	n.i.	1
	Weaned	Individual – Injectable	12 mg/kg; 1 day	1
		Individual – Injectable	n.i.	2
	Growing	Individual – Injectable	7.5 mg/kg; 1 day	1
		Individual – Injectable	Dosage n.i.; 3 days	1
	Finishing	Individual – Injectable	n.i.	3
**Norfloxacin**				**5 (2.7%)**
Prophylaxis	Gestating	Group – feed	400 g/ton feed; 10/45 days	1
	Lactating	Group – feed	200 g/ton feed; continuous	1
	Finishing	Group – water	Dosage n.i.; 15/15 days	1
Metaphylaxis	Finishing	Group – water	12 mg/kg; Limited; days n.i.	2
**Lincosamide (Lincomycin)**				**4 (2.2%)**
Prophylaxis	Finishing	Group – feed	100 g/ton feed; 10 days	1
		Group – feed	Dosage n.i.; 15 days	1
Metaphylaxis	Finishing	Group – feed	Dosage n.i.; 7 days	2
**Macrolide**				**37 (20.1%)**
**Erythromycin**				**1 (0.5%)**
Prophylaxis	Weaned	Group – feed	200 g/ton feed; continuous	1
**Tilmicosin**				**13 (7.1%)**
Prophylaxis	Lactating	Group – feed	200 g/ton feed; continuous	2

	Weaned	Group – water	15 mg/kg; 7 days	1
	Growing	Group – feed	240 g/ton feed; continuous	2
	Finishing	Group – feed	240 g/ton feed; continuous	1
		Group – feed	400 g/ton feed; 15/15 days	1
Metaphylaxis	Weaned	Group – feed	200 g/ton feed; continuous	1
	Finishing	Group – feed	Dosage n.i.; 10 days	2
		Group – feed	Dosage n.i.; 15 days	2
Treatment	Lactating	Group – feed	200 g/ton feed; continuous	1
**Tylosin**				**15 (8.2%)**
Prophylaxis	Gestating	Group – feed	100 g/ton feed; 15 days	1
		Group – feed	Dosage n.i.; 7/90 days	1
	Lactating	Group – feed	Dosage n.i.; 7 days	1
	Finishing	Group – feed	88 g/ton feed; regimen n.i.	1
		Group – feed	Dosage n.i.; 7 days	1
Metaphylaxis	Growing	Group – feed	Dosage n.i.; 15 days	2
	Finishing	Group – feed	Dosage n.i.; 15 days	2
Treatment	Gestating	Individual – Injectable	10 mg/kg; 3 days	1
	Weaned	Individual – Injectable	n.i.	1
	Finishing	Individual – Injectable	10 mg/kg; 3 days	1
		Individual – Injectable	Dosage n.i.; 7 days	1
		Individual – Injectable	n.i.	2
**Tylvalosin**				**3 (1.6%)**
Prophylaxis	Gestating	Group – feed	45 g/ton feed; 15 days	1
	Weaned	Group – feed	65 g/ton feed; 10 days	1
		Group – water	5 mg/kg; 7 days	1
**Tulathromycin**				**5 (2.7%)**
Treatment	Lactating	Individual – Injectable	n.i.	1
	Weaned	Individual – Injectable	2,5 mg/kg; Limited; days n.i.	1
		Individual – Injectable	n.i.	1
	Finishing	Individual – Injectable	n.i.	2
**Narrow-spectrum Penicillin (Penicilin G)**				**6 (3.3%)**
Treatment	Gestating	Individual – Injectable	15000 UI/kg; 3 days	1
		Individual – Injectable	n.i.	1
	Lactating	Individual – Injectable	Dosage n.i.; 2 y 3 days	2
	Weaned	Individual – Injectable	15000 UI/kg; Limited; days n.i.	1
	Finishing	Individual – Injectable	15000 UI/kg; Limited; days n.i.	1
**Pleuromutilin (Tiamulin)**				**20 (10.9%)**
Prophylaxis	Gestating	Group – feed	300 g/ton feed; continuous	1
		Group – feed	100 g/ton feed; 7/21 days	1
		Group – feed	300 g/ton feed; 15/30 days	1
		Group – feed	100 g/ton feed; 15/45 days	1
		Group – feed	200 g/ton feed; regimen n.i.	1
	Lactating	Group – feed	100 g/ton feed; continuous	1
	Growing	Group – feed	184 g/ton feed; 15 days	1
		Group – feed	40 g/ton feed; 15 days	1
		Group – feed	100 g/ton feed; 15/15 days	1

	Finishing	Group – feed	100 y 200 g/ton feed; continuous	2
		Group – feed	100 y 400 g/ton feed; 15/15 days	2
		Group – feed	165 g/ton feed; 10/10 days	1
		Group – feed	160 g/ton feed; 21/30 days	1
		Group – feed	100 g/ton feed; regimen n.i.	1
Metaphylaxis	Growing	Group – feed	200 g/ton feed; 15/15 days	1
	Finishing	Group – feed	150 g/ton feed; 15/15 days	1
		Group – feed	Dosage n.i.; 15 days	2
**Polypeptide (Bacitracin)**				**1 (0.5%)**
Growth promotion	Finishing	Group – feed	n.i.	1
**Sulfonamides**				**3 (1.6%)**
**Sulfamethoxazole/Trimethoprim**				**1 (0.5%)**
Treatment	Finishing	Individual – Injectable	n.i.	1
**Sulfamethazine**				**2 (1.1%)**
Prophylaxis	Weaned	Group – water	Dosage n.i.; 7 days	1
Treatment	Lactating	Individual – Injectable	n.i.	1
**Tetracyclines**				**20 (10.9%)**
**Chlortetracycline**				**14 (7.6%)**
Prophylaxis	Gestating	Group – feed	400 g/ton feed; 15 days	1
		Group – feed	400 g/ton feed; 7/21 days	1
		Group – feed	300 g/ton feed; regimen n.i.	1
	Lactating	Group – feed	400 g/ton feed; continuous	1
	Growing	Group – feed	100 y 400 g/ton feed; 15 days	2
		Group – feed	400 g/ton feed; regimen n.i.	1
	Finishing	Group – feed	500 y 600 g/ton feed; continuous	2
		Group – feed	440 g/ton feed; 10/10 days	1
		Group – feed	400 g/ton feed; 15/15 days	1
		Group – feed	120 g/ton feed; regimen n.i.	1
Metaphylaxis	Finishing	Group – feed	600 g/ton feed; 15/15 days	1
Treatment	Finishing	Individual – Injectable	n.i.	1
**Doxycycline**				**3 (1.6%)**
Prophylaxis	Weaned	Group – water	10 mg/kg; 7 days	1
	Finishing	Group – water	10 mg/kg; 7/21 days	1
Metaphylaxis	Growing	Group – feed	300 g/ton feed; 15/15 days	1
**Oxytetracycline**				**3 (1.6%)**
Treatment	Weaned	Individual – Injectable	Dosage n.i.; 2 days	1
		Individual – Injectable	n.i.	1
	Finishing	Individual – Injectable	n.i.	1
**n.i.**				**1 (0.5%)**
Prophylaxis	Finishing	Group – feed	n.i.	1

n.i.: not informed. Dosages refer to the amount of active ingredient expressed as mg or g per kg of animal body weight or per ton of feed, respectively. In intermittent dosing regimens, the duration of treatment and the interval before the next cycle are indicated (X/Y: for X days every Y days).
