# Supplementary material for: Antimicrobial Use and Epidemiological Resistance Profiles of Commensal Escherichia coli from Swine Farms in Córdoba, Argentina
Source: Antibiotics (Basel). 2026 Jan 15;15(1):86. doi: 10.3390/antibiotics15010086 (PMC12837807; doi:10.3390/antibiotics15010086)
Supplement: Supplementary file 1 [file antibiotics-15-00086-s001.zip › Table S2.pdf]

Table S2: Detailed MDR (NWT) profiles in *Escherichia coli*: combinations of affected antimicrobial classes and agents.

Number of antimicrobial classes affected (NWT)	Isolates (n)	Isolates / Categories (n)					Farms (n)
		Gestating	Lactating	Weaned	Growing	Finishing	
**None**							
-	2	1	0	1	0	0	1
**One class**							
AMP	3	0	1	0	1	1	1
SMX	2	0	0	0	0	2	1
SMX, TMP	1	0	1	0	0	0	1
TET	1	0	0	1	0	0	1
TMP	2	1	0	0	0	1	2
**Two classes**							
AMP, CHL	7	3	1	1	1	1	3
AMP, CTX, CHL	1	0	0	0	0	1	1
AMP, MEM, CHL	1	0	0	0	1	0	1
AMP, TET	2	0	0	0	0	2	2
CHL, CIP	1	1	0	0	0	0	1
CHL, GEN	1	0	0	0	1	0	1
TET, CHL	2	0	1	0	1	0	2
TET, SMX	1	0	0	0	1	0	1
TET, SMX, TMP	6	3	2	1	0	0	3
TET, TMP	2	0	1	1	0	0	1
**Three classes**							
AMP, CHL, SMX	2	0	2	0	0	0	2
AMP, CHL, SMX, TMP	4	1	2	1	0	0	3
AMP, CHL, TMP	2	0	0	0	2	0	1
AMP, CTX, CHL, SMX	2	0	1	1	0	0	1
AMP, CTX, CHL, SMX, TMP	1	0	0	0	0	1	1
AMP, CTX, MEM, CHL, SMX, TMP	1	0	1	0	0	0	1
AMP, CTX, TET, CHL	2	2	0	0	0	0	2
AMP, CTX, TET, SMX, TMP	2	0	2	0	0	0	2
AMP, TET, CIP	1	1	0	0	0	0	1
AMP, TET, CHL	23	7	2	7	3	4	11
AMP, TET, SMX	1	0	0	0	0	1	1
AMP, TET, SMX, TMP	6	2	2	2	0	0	5
AMP, TET, TMP	2	1	0	1	0	0	2
TET, CHL, CIP	2	1	1	0	0	0	1
TET, CHL, SMX	3	1	1	0	0	1	3
TET, CHL, SMX, TMP	3	2	0	0	1	0	3
TET, CHL, TMP	1	0	0	0	1	0	1

**Four classes**							
AMP, CHL, CIP, SMX	2	2	0	0	0	0	1
AMP, CHL, CIP, SMX, TMP	4	0	2	0	2	0	1
AMP, CTX, TET, CHL, CIP	2	0	0	0	1	1	2
AMP, CTX, TET, CHL, SMX	6	2	1	0	2	1	4
AMP, CTX, TET, CHL, SMX, TMP	3	0	3	0	0	0	3
AMP, CTX, TET, CHL, TMP	2	0	0	2	0	0	1
AMP, MEM, TET, CHL, CIP	1	0	0	0	0	1	1
AMP, MEM, TET, CHL, SMX	1	0	0	0	1	0	1
AMP, TET, CIP, SMX	1	0	1	0	0	0	1
AMP, TET, CIP, SMX, TMP	2	0	0	1	1	0	2
AMP, TET, CIP, TMP	1	0	0	1	0	0	1
AMP, TET, CHL, CIP	19	4	4	3	3	5	9
AMP, TET, CHL, COL	3	0	0	3	0	0	1
AMP, TET, CHL, GEN	7	0	1	1	3	2	3
AMP, TET, CHL, SMX	41	15	5	4	8	9	12
AMP, TET, CHL, SMX, TMP	38	7	7	3	9	12	8
AMP, TET, CHL, TMP	10	1	1	0	4	4	5
AMP, TET, GEN, TMP	1	0	0	0	0	1	1
CTX, TET, CHL, CIP	1	1	0	0	0	0	1
CTX, TET, CHL, SMX	1	0	0	0	1	0	1
TET, CHL, CIP, SMX	2	0	1	1	0	0	1
TET, CHL, CIP, SMX, TMP	1	1	0	0	0	0	1
**Five classes**							
AMP, CHL, CIP, COL, SMX, TMP	1	0	1	0	0	0	1
AMP, CTX, MEM, TET, CHL, SMX	1	0	1	0	0	0	1
AMP, CTX, TET, CHL, CIP, GEN	1	1	0	0	0	0	1
AMP, CTX, TET, CHL, CIP, SMX, TMP	13	1	2	6	2	2	5
AMP, CTX, TET, CHL, COL, SMX	1	0	0	1	0	0	1
AMP, CTX, TET, CHL, GEN, SMX, TMP	2	0	0	1	0	1	2
AMP, MEM, TET, CHL, CIP, SMX	7	2	1	0	2	2	5
AMP, MEM, TET, CHL, CIP, SMX, TMP	2	0	0	1	0	1	2
AMP, MEM, TET, CHL, CIP, TMP	2	1	0	0	0	1	2
AMP, TET, CIP, GEN, SMX	2	0	0	1	1	0	2
AMP, TET, CIP, GEN, TMP	1	0	0	0	0	1	1
AMP, TET, CHL, CIP, COL	2	0	1	1	0	0	2
AMP, TET, CHL, CIP, GEN	4	0	0	3	0	1	2
AMP, TET, CHL, CIP, SMX	59	12	13	8	13	13	11
AMP, TET, CHL, CIP, SMX, TMP	36	7	5	3	14	7	13
AMP, TET, CHL, CIP, TMP	8	2	1	2	2	1	5
AMP, TET, CHL, COL, SMX, TMP	2	0	0	2	0	0	1

AMP, TET, CHL, GEN, COL	1	0	0	0	0	1	1
AMP, TET, CHL, GEN, SMX	4	0	1	0	1	2	3
AMP, TET, CHL, GEN, SMX, TMP	2	0	0	1	1	0	2
TET, CHL, CIP, GEN, TMP	1	1	0	0	0	0	1
**Six classes**							
AMP, CTX, TET, CHL, CIP, GEN, SMX	1	0	1	0	0	0	1
AMP, CTX, TET, CHL, CIP, GEN, SMX, TMP	5	0	3	2	0	0	3
AMP, CTX, TET, CHL, GEN, COL, SMX	1	0	1	0	0	0	1
AMP, TET, CHL, CIP, COL, SMX, TMP	3	1	0	1	1	0	3
AMP, TET, CHL, CIP, COL, TMP	1	1	0	0	0	0	1
AMP, TET, CHL, CIP, GEN, COL	1	0	0	1	0	0	1
AMP, TET, CHL, CIP, GEN, SMX	8	1	1	2	2	2	4
AMP, TET, CHL, CIP, GEN, SMX, TMP	9	1	0	4	3	1	6
AMP, TET, CHL, CIP, GEN, TMP	10	2	3	2	2	1	2
**Seven classes**							
AMP, CTX, TET, CHL, CIP, GEN, COL, SMX, TMP	1	0	0	1	0	0	1
AMP, TET, CHL, CIP, GEN, COL, SMX	1	0	0	0	0	1	1
AMP, TET, CHL, CIP, GEN, COL, TMP	2	0	2	0	0	0	1

AMP: ampicillin; TET: tetracycline; CHL: chloramphenicol; SMX: sulfamethoxazole; CIP: ciprofloxacin; TMP: trimethoprim; GEN: gentamicin; CTX: cefotaxime; COL: colistin; MEM: meropenem.
